# Supplementary material for: Determinants of intentions to monitor antihypertensive medication adherence in Irish community pharmacy: a factorial survey
Source: BMC Fam Pract. 2019 Sep 13;20:131. doi: 10.1186/s12875-019-1016-6 (PMC6744667; doi:10.1186/s12875-019-1016-6)
Supplement: Supplementary file 1 — Questionnaire. The questionnaire completed by survey respondents (PDF 345 kb) [file 12875_2019_1016_MOESM1_ESM.pdf]

Questionnaire

1 Start

Welcome to the survey.

Before completing this survey please ensure that you have read the Information Leaflet, which you have been provided with, which contains important information. We also would like to ask you your consent to aggregate your data with others for future use in a research paper.

Informed Consent

*I have read and understood the **Information Leaflet** about this research project. The information provided has been sufficient and I have been able to ask questions, all of which have been answered to my satisfaction. I understand that I don't have to provide consent.*

○ Yes

Data Protection

I give permission for study material/data to be stored for possible future research related to the current study without further consent being required subject to research ethics committee approval. No individual will be identifiable from stored information.

○ Yes

○ No

2 Eligibility Check

Eligibility check

Do you currently work as a **community pharmacist** in the ***Republic of Ireland***?

○ Yes

○ No

3 MMAM

This first section contains a list of statements other pharmacists have previously used to describe their experiences in monitoring patients on medication for long-term conditions.

Please read each of the following statements and indicate, depending on your level of agreement with the statement, whether you strongly agree (6), moderately agree (5), slightly agree (4), slightly disagree (3), moderately disagree (2) and strongly disagree (1).

	Strongly Disagree	Moderately Disagree	Slightly Disagree	Slightly Agree	Moderately Agree	Strongly Agree
I do not have time to talk to everyone who is late collecting repeat medication for long-term conditions.	○	○	○	○	○	○
Talking to patients about non-adherence makes me uncomfortable.	○	○	○	○	○	○
Pharmacists should routinely follow-up with patients to see how their long-term medication is working.	○	○	○	○	○	○
I become concerned when someone is late obtaining repeat medication for long-term conditions.	○	○	○	○	○	○
It is my responsibility to ensure the patient's long-term medication is effective.	○	○	○	○	○	○
I feel confrontational when I bring up non-adherence	○	○	○	○	○	○

with patients.

	Strongly Disagree	Moderately Disagree	Slightly Disagree	Slightly Agree	Moderately Agree	Strongly Agree
It is a good idea to ask patients about medication side effects.	○	○	○	○	○	○
Interacting with patients about their long-term medication is a satisfying part of my job.	○	○	○	○	○	○
I am able to positively impact the health of patients taking medication for chronic illnesses.	○	○	○	○	○	○
It is my responsibility to ensure that patients are not having unnecessary side effects.	○	○	○	○	○	○
I see so many people on the same drugs that it is hard for any of my patients to stand out.	○	○	○	○	○	○
Patients rarely want to discuss their long-term medication with me.	○	○	○	○	○	○
My pharmacy is too busy to get to know individual patients.	○	○	○	○	○	○
Patients do not want me asking about their long-term medication.	○	○	○	○	○	○
I feel hurried when I talk to patients about long-term medication.	○	○	○	○	○	○

4 Short Scenarios

The next section consists of 6 short vignettes or scenarios. Each vignette will describe a familiar dispensing scenario. The patients described in the scenarios are ***all older patients*** who are at least **65 years old**.

The vignette will then be followed by three questions asking you to rate whether you would perform a specific action in each scenario. You are asked to read each scenario and answer the corresponding questions before proceeding to the next scenario. Please be honest and remember that your responses are anonymous.

5.1 Vig_1

#u_vig_1#

In your current work practice, if presented with this scenario, how likely would you:

Responses: scale 1-10 (1 being the least likely and 10 most likely)

Examine this patient’s dispensing records to assess adherence to antihypertensive medication over the previous months

Least Likely — Most Likely

Question this patient about their adherence to antihypertensive medication

Least Likely — Most Likely

Explore beliefs about antihypertensive medication that may influence this patient's adherence

Least Likely Most Likely

5.2 Vig_2

#u_vig_2#

In your current work practice, if presented with this scenario, how likely would you:

Responses: scale 1-10 (1 being the least likely and 10 most likely)

Examine this patient's dispensing records to assess adherence to antihypertensive medication over the previous months

Least Likely Most Likely

Question this patient about their adherence to antihypertensive medication

Least Likely Most Likely

Explore beliefs about antihypertensive medication that may influence this patient's adherence

Least Likely Most Likely

5.3 Vig_3

#u_vig_3#

In your current work practice, if presented with this scenario, how likely would you:

Responses: scale 1-10 (1 being the least likely and 10 most likely)

Examine this patient's dispensing records to assess adherence to antihypertensive medication over the previous months

Least Likely Most Likely

Question this patient about their adherence to antihypertensive medication

Least Likely Most Likely

Explore beliefs about antihypertensive medication that may influence this patient's adherence

Least Likely Most Likely

5.4 Vig_4

#u_vig_4#

In your current work practice, if presented with this scenario, how likely would you:

Responses: scale 1-10 (1 being the least likely and 10 most likely)

Examine this patient's dispensing records to assess adherence to antihypertensive medication over the previous months

Least Likely Most Likely

Question this patient about their adherence to antihypertensive medication

Least Likely Most Likely

Explore beliefs about antihypertensive medication that may influence this patient's adherence

Least Likely Most Likely

5.5 Vig_5

#u_vig_5#

In your current work practice, if presented with this scenario, how likely would you:

Responses: scale 1-10 (1 being the least likely and 10 most likely)

Examine this patient's dispensing records to assess adherence to antihypertensive medication over the previous months

Least Likely Most Likely

Question this patient about their adherence to antihypertensive medication

Least Likely Most Likely

Explore beliefs about antihypertensive medication that may influence this patient's adherence

Least Likely Most Likely

5.6 Standard_vig

You are dispensing a repeat prescription for a new elderly patient, Mr. Gallagher, who has been attending the pharmacy for three months now. The end of the month is approaching and you are conscious of completing the monthly claim. Mr. Gallagher has phoned the prescription in and will have his daughter collect it later; his prescription has 5 items and you see from the computer he is 5 days late collecting his repeat prescription. He has been on medication to treat ***Hypertension*** for 6 months, and has expressed doubts about the need to take antihypertensive medication. The pharmacy is short-staffed today and there are 3 other patients waiting for prescriptions. While dispensing this prescription another patient has asked to speak to the pharmacist.

In your current work practice, if presented with this scenario, how likely would you:

Responses: scale 1-10 (1 being the least likely and 10 most likely)

Examine this patient's dispensing records to assess adherence to

antihypertensive medication over the previous months

Least Likely — Most Likely

Question this patient about their adherence to antihypertensive medication

Least Likely — Most Likely

Explore beliefs about antihypertensive medication that may influence this patient's adherence

Least Likely — Most Likely

6 Final Questions

In this third section of the survey you will be presented with one of the vignettes that you have assessed in the previous section and you will be asked to reflect on how this scenario mirrors real-life practice. You will also be asked to rate your agreement with six statements about practice norms in pharmacy.

6.1 Reflections

Please re-read the scenario that you responded to earlier and consider how realistic you think this scenario is in relation to your everyday practice.

You are dispensing a repeat prescription for a new elderly patient, Mr. Gallagher, who has been attending the pharmacy for three months now. The end of the month is approaching and you are conscious of completing the monthly claim. Mr. Gallagher has phoned the prescription in and will have his daughter collect it later; his prescription has 5 items and you see from the computer he is 5 days late collecting his repeat prescription. He has been on medication to treat ***Hypertension*** for 6 months, and has expressed doubts about the need to take antihypertensive medication. The pharmacy is short-staffed today and there are 3 other patients waiting for prescriptions. While dispensing this prescription another patient has asked to speak to the pharmacist.

Responses: scale 1-10 (1 being very unrealistic and 10 very realistic)

How realistic do you find this scenario in relation to your own practice?

Very unrealistic — Very realistic

6.2 Practice norms

Please rate your agreement with the following statements:

Other pharmacists examine their patient's dispensing records to assess adherence to antihypertensive medication over the previous months.

7 Level scale: False - True

False — Neither True nor False — True

For me examining my patient's dispensing records to assess adherence to antihypertensive medication over the previous months is:

7 Level scale: Difficult-Easy

Difficult — Neither Easy nor Difficult — Easy

Other pharmacists ask their patients questions about their adherence to antihypertensive medication.

7 Level scale: False-True

False | Neither True nor False | True

For me asking my patients questions about their adherence to antihypertensive medication is:

7 Level scale: Difficult-Easy

Difficult | Neither Easy nor Difficult | Easy

Other pharmacists discuss medication beliefs that influence antihypertensive medication with their patients.

7 Level scale: False-True

False | Neither True nor False | True

For me discussing medication beliefs that influence antihypertensive medication with my patients is:

7 Level scale: Difficult-Easy

Difficult | Neither Easy nor Difficult | Easy

GPs in my locality think that I should assess patients' antihypertensive medication adherence when dispensing repeat prescriptions.

7 Level scale: Should not - Should

Should not | Neither should nor should not | Should

As a pharmacist, it is expected that I assess patients' antihypertensive medication adherence when dispensing repeat prescriptions.

7 Level scale: False - True

False | Neither True nor False | True

Patients would approve that I assess their antihypertensive medication adherence when dispensing repeat prescriptions.

7 Level scale: Disapprove - Approve

Disapprove | Neither Approve nor Disapprove | Approve

7 Descriptives

You are almost finished!

The final section of the survey will ask you questions about you and your place of work. The purpose of this section is to gauge your working environment.

Please remember that your responses are **anonymous.**

8 Pharmacist and Pharmacy Practice Details

Are you..?

○ Male ○ Female

When did you qualify as a pharmacist?

	Month	Year
Month/Year	January<br>Februar<br>y March<br>April<br>May<br>June<br>July<br>August<br>September<br>October<br>November<br>December	

1960
1961
1962
1963
1964
1965
1966
1967
1968
1969
1970
1971
1972
1973
1974
1975
1976
1977
1978
1979
1980
1981
1982
1983
1984
1985
1986
1987
1988
1989
1990
1991
1992
1993
1994
1995
1996
1997
1998
1999
2000
2001
2002
2003
2004
2005
2006
2007
2008
2009
2010
2011
2012
2013
2014
2015
2016
2017

What type of community pharmacy do you work in?

- ◯ Independent
- ◯ Chain
- ◯ Part of a 'symbol' group
- ◯ I work across various pharmacy types

How would you define your role as a community pharmacist?

Tick all that apply

- ☐ Support
- ☐ Supervising
- ☐ Superintendent
- ☐ Store Manager
- ☐ Owner
- ☐ Locum
- ☐ Relief

Where is your pharmacy located?

If you work across multiple pharmacies please select type where you spend greatest proportion of time

- ○ High Street/Town Centre
- ○ Shopping Centre/Retail Centre
- ○ Residential Area
- ○ Rural
- ○ Other

On a typical day how many items do you dispense in your pharmacy?

Estimate to the nearest multiple of 5

How many pharmacists do you typically work with?

Please indicate the total number of pharmacists, excluding yourself. Please include part-time pharmacists as a ratio of 1, where 1 is equivalent to 5 days a week. For example working with a part-time pharmacist who works half-days is equivalent to 0.5

How many technicians/dispensers do you typically work with?

Please indicate the total number including part-time technicians as a ratio of 1, where 1 is equivalent to 5 days a week. For example working with a part-time technician who works half-days is equivalent to 0.5.

How many non-dispensary staff do you typically work with?

Please indicate the total number, including part-time staff as a ratio of 1, where 1 is equivalent to 5 days a week. For example working with a non-dispensary part-time staff member who works half-days is equivalent to 0.5.

Typically how many hours a week do you work in **community** pharmacy?

What **proportion of this time** is devoted to adminstrative tasks? (Score 0-100, do not use %)

Administrative tasks include processing claims (e.g verifying), applying for reimbursement approval (e.g. hardship, phased dispensing), self-assessment reports etc

Does your pharmacy provide clinical pharmacy service for the monitoring of blood pressure?

Tick all that apply

- ☐ 24 hr Ambulatory Monitoring
- ☐ Formal Blood Pressure Measurement
- ☐ Informal Blood Pressure Measurement
- ☐ No Blood Pressure Measurement

Does your pharmacy provide other clinical pharmacy services?

(e.g cholesterol/diabetes screening, vaccinations, smoking cessation)

- ○ Yes (please specify)
- ○ No

9 Feedback

Before you finish the survey, are there any comments you would like to make?

Would you like to be entered into the draw to win a €100 or €50 gift card?

Please enter your email address below.

10 Final page

You are now finished the questionnaire!

Thank You.
