# Supplementary material for: Determinants of intentions to monitor antihypertensive medication adherence in Irish community pharmacy: a factorial survey
Source: BMC Fam Pract. 2019 Sep 13;20:131. doi: 10.1186/s12875-019-1016-6 (PMC6744667; doi:10.1186/s12875-019-1016-6)
Supplement: Supplementary file 2 — Study information leaflet (PDF 384 kb) [file 12875_2019_1016_MOESM2_ESM.pdf]

Information Leaflet

RCSI SCHOOL OF PHARMACY

Study title: Factors influencing pharmacists likelihood to assess anti-hypertensive medication adherence in older adults: A factorial survey

**Principal investigator's name:**	**Paul Dillon MPSI**
**Principal investigator's title:**	**PhD Student**
**Telephone number of principal investigator:**	**01 402 5130**
**Co-investigator's name:**	**Dr Gráinne Cousins**
**Co-investigator's title:**	**Lecturer in Pharmacy**

As a practicing community pharmacist you are being invited to take part in a research study. Before you decide whether or not you wish to take part, you should read the information provided below carefully.

Why is this study being done?

This study is being carried out to gain insight to the attitudes and beliefs of community pharmacists towards medication adherence and barriers to pharmacist interactions with older patients who may be at risk of poor antihypertensive adherence. Your survey responses will be aggregated with other pharmacists' data to produce a research paper that will form part of our research Programme which is focused on developing community pharmacy interventions to improve patient medication adherence.

Who is organising and funding this study?

This research is being carried out in the Royal College of Surgeons in Ireland. Paul Dillon is the contact person for this study. His contact details are listed below.

Why am I being asked to take part?

As a community pharmacist working in the Republic of Ireland we need your insight to assess the challenges of medication adherence and to inform future research investigating expanded roles of community pharmacists including a pharmacist led intervention to improve medication adherence.

How will the study be carried out?

You will receive a link to the survey which consists of four main sections:

1. Questions about your current pharmacy practice, such as time since you qualified, type of pharmacy you work in and busyness of the pharmacy;
2. A questionnaire known as the Medication Monitoring Attitude Measure (MMAM), which includes a list of statements other pharmacists have previously made regarding monitoring of patients with long-term medication
3. A series of six clinical scenarios known as vignettes about a patient attending the pharmacy to obtain repeat medication for hypertension. In response to each scenarios you will be asked questions on your likelihood to **a)** Examine this patient's dispensing records to assess long-term antihypertensive medication adherence, **b)** Question this patient about antihypertensive medication adherence, and **c)** Explore beliefs about antihypertensive medication that may influence this patient's adherence
4. A short series of questions about current norms in current pharmacy practice

Note there is no right or wrong answer – it is your opinion we want to hear. The online survey tool ***Unipark*** will be used to create and complete the survey, which can securely collect the data for analysis. Your response will be anonymous and we will not collect any data that may identify you.

What are the benefits?

There are no direct benefits to you in taking part in the study.

What are the risks?

There are no identified risks to you in taking part in the study.

Is the study confidential?

All your information will remain strictly confidential and your responses will be anonymous. After the survey is completed, all data will be encrypted and stored on a single password protected computer in the Royal College of Surgeons in Ireland and only the lead researcher, Mr Paul Dillon, will have access to the data. The data will be securely stored and password protected in the Royal College of Surgeons for a maximum of 5-7 years.

We will not collect identifiable information from you and you will not be identifiable from any research papers arising from this study.

Where can I get further information?

If you have any further questions about the study or if you want to opt out of the please contact:

Name: Mr Paul Dillon
Address: School of Pharmacy, Royal College of Surgeons in Ireland, Dublin 2.
Email: pauldillon@rcsi.ie
Tel: 01-4025130 (Mon-Fri 9-6pm)
