# Supplementary material for: Determinants of intentions to monitor antihypertensive medication adherence in Irish community pharmacy: a factorial survey
Source: BMC Fam Pract. 2019 Sep 13;20:131. doi: 10.1186/s12875-019-1016-6 (PMC6744667; doi:10.1186/s12875-019-1016-6)
Supplement: Supplementary file 3 — Sample size. Detailed description of procedure to estimate sample size for the survey (ZIP 213 kb) [file 12875_2019_1016_MOESM3_ESM.zip › pharmacist_factorial_survey_finalR3.docx]

# Determinants of intentions to monitor antihypertensive medication adherence in Irish community pharmacy: a factorial survey

**Authors**

*Paul Dillon, PhD student, School of Pharmacy, RCSI, St. Stephen’s Green, Dublin 2, Ireland. pauldillon@rcsi.ie

Dr Ronald McDowell, Postdoctoral Research Fellow in Biostatistics, HRB Centre for Primary Care Research, RCSI, St. Stephen’s Green, Dublin 2, Ireland. ronaldmcdowell@rcsi.ie

Prof Susan M Smith, Professor of Primary Care Medicine, Department of General Practice and HRB Centre for Primary Care Research, RCSI, St. Stephen’s Green, Dublin 2, Ireland. susansmith@rcsi.ie

Prof Paul Gallagher, Head of School, School of Pharmacy, RCSI, St. Stephen’s Green, Dublin 2, Ireland. pgallagher@rcsi.ie

Dr Gráinne Cousins, Senior Lecturer, School of Pharmacy, RCSI, St. Stephen’s Green, Dublin 2, Ireland. gcousins@rcsi.ie

*Corresponding author

# Abstract

## Background

Community pharmacy represents an important setting to identify patients who may benefit from an adherence intervention, however it remains unclear whether it would be feasible to monitor antihypertensive adherence within the workflow of community pharmacy. The aim of this study was to identify facilitators and barriers to monitoring antihypertensive medication adherence of older adults at the point of repeat dispensing.

## Methods

We undertook a factorial survey of Irish community pharmacists, guided by a conceptual model adapted from the Theory of Planned Behaviour (TPB). Respondents completed four sections, 1) five factorial vignettes (clinical scenario of repeat dispensing), 2) a medication monitoring attitude measure, 3) subjective norms and self-efficacy questions, and 4) demographic and workplace questions. Barriers and facilitators to adherence monitoring behaviour were identified in factorial vignette analysis using multivariate multilevel linear modelling, testing the effect of both contextual factors embedded within the vignettes (section 1), and respondent-level factors (sections 2-4) on likelihood to perform three adherence monitoring behaviours in response to the vignettes.

## Results

Survey invites (n=1,543) were sent via email and 258 completed online survey responses were received; two-thirds of respondents were women, and one-third were qualified pharmacists for at least 15 years. In factorial vignette analysis, pharmacists were more inclined to monitor antihypertensive medication adherence by examining refill-patterns from pharmacy records than asking patients questions about their adherence or medication beliefs. Pharmacists with more positive attitudes towards medication monitoring and normative beliefs that other pharmacists monitored adherence, were more likely to monitor adherence. Contextual factors also influenced pharmacists’ likelihood to perform the three adherence monitoring behaviours, including time-pressures and the number of days late the patient collected their repeat prescription. Pharmacists’ normative beliefs and the number of days late the patient collected their repeat prescription had the largest quantitative influence on responses.

## Conclusions

This survey identified that positive pharmacist attitudes and normative beliefs can facilitate adherence monitoring within the current workflow; however contextual time-barriers may prevent adherence monitoring. Future research should consider these findings when designing a pharmacist-led adherence intervention to be integrated with current pharmacy workflow.

## Keywords

adherence interventions, community pharmacy, factorial survey, medication adherence, medication monitoring, pharmacist attitudes, Republic of Ireland, time-pressures

# Background

Poor adherence to antihypertensive medication is estimated at 40% [1]. Numerous interventions to improve adherence to antihypertensive medication have proven to be effective, including technical approaches such as reducing the number of daily doses, reminder interventions for forgetful patients, and behavioural approaches to modify patient beliefs [2-6]. However, successful adherence interventions tend to be complex, involving multiple components and frequent interactions with patients [2-6]. The resulting complexity has been highlighted as a barrier to the successful implementation of adherence interventions in practice [3, 5]. Stratifying appropriate patients for adherence interventions may aide the feasibility in practice, as fewer resources are required to deliver the intervention, while tailoring interventions to the patient specific barrier appears to be more effective than general interventions [7-11]. For example pharmacy refill metrics have been used to target patients with poor adherence, and patient-specific barriers, such as beliefs about medication have been evaluated using questionnaires, to tailor the relevant intervention component [7]. There is, however an absence of studies investigating the feasibility of identifying poor adherence in practice [12].

Given that most patients prescribed antihypertensive medication attend a pharmacy at least once a month [13], community pharmacy represents an important setting to identify patients who may benefit from an adherence intervention and enable the targeting and tailoring of adherence interventions [12, 14]. Pharmacists have access to dispensing records to allow assessment of refill adherence while regular contact can facilitate discussion with patients on their adherence behaviour and barriers [12, 14]. However, challenges to pharmacist led-interventions include time barriers, inter-professional working arrangements, and absence of reimbursement models outside of a research setting [3, 15-24]. It remains unclear whether it would be feasible and compatible to identify poor adherence within the workflow of community pharmacy. Furthermore, pharmacist attitudes towards a proposed intervention have been highlighted as an important facilitator of an intervention’s implementation [25, 26]. Medication monitoring attitudes held by community pharmacists have been identified as a significant determinant of adherence monitoring behaviours during repeat dispensing [18, 27]. Thus, a pharmacist’s perception of their role and responsibility, and perception of their work environment may also influence the feasibility of a structured adherence-monitoring programme [18, 27].

Due to the absence of studies investigating the feasibility of monitoring adherence within the workflow of community pharmacy we undertook a factorial survey of Irish community pharmacists with 1) the objectives to elicit pharmacist beliefs regarding monitoring of antihypertensive adherence, and 2) to identify facilitators and barriers to monitoring antihypertensive medication adherence of older adults at the point of repeat dispensing. The factorial survey was guided by a conceptual model adapted from the Theory of Planned Behaviour (TPB), which has been highlighted as a useful framework to understand barriers and facilitators to extended pharmacist roles in practice [25].

# Methods

## Survey Overview

A factorial survey of community pharmacists from the Republic of Ireland was undertaken in August 2017 (n=258). A sampling frame of potential participants was identified with permission from the Pharmaceutical Society of Ireland (PSI), who maintain the register of pharmacists in the Republic of Ireland. A simple random sample (n=1,543) of potential respondents were contacted via email addresses provided by the PSI and were sent a unique password protected web-link to complete the survey online. Respondents completed four sections, 1) five factorial vignettes, 2) a medication monitoring attitude measure, 3) subjective norms and self-efficacy questions, and 4) demographic and workplace questions (Appendix 1). Pharmacists’ beliefs regarding adherence monitoring were elicited in sections 2 and 3 of the survey. Barriers and facilitators to adherence monitoring were identified in factorial vignette analysis, testing the effect of both contextual factors embedded within the vignettes (section 1), and respondent-level factors (sections 2-4) on likelihood to perform three adherence monitoring behaviours in response to the vignettes.

Respondents were provided with an information study leaflet (Appendix 2) and provided informed consent before completing the survey. Ethical approval was granted by the Research and Ethics Committee (REC) at the Royal College of Surgeons in Ireland (RCSI) (REC application 1356/2017).

## Survey Framework

A conceptual model was adapted from the TPB including multilevel contextual factors, to guide this survey to identify barriers and facilitators to antihypertensive adherence monitoring behaviours during repeat dispensing in a community pharmacy (Figure 1). The TPB describes the influence of an individual’s behavioural, normative and control beliefs on their behaviour [28]. In general, more favourable attitudes and subjective norms towards the behaviour, and greater perceived behavioural control, result in stronger behavioural intentions [28]. The TPB has been shown to be useful in predicting significant proportions of behavioural intention across a wide range of behaviours [29], but also to understand healthcare professional’s clinical behaviours including those of pharmacists [25, 30, 31]. The TPB framework served as a guide, to ensure important constructs potentially influencing behaviour were evaluated; however social, organisational, political and economic factors, must also be included in theoretical models that seek to evaluate the feasibility of implementing a new clinical service such as an adherence intervention in community pharmacy [30, 32]. The current study was designed using items from pre-existing questionnaires, from qualitative discussions with academic pharmacists experienced in community pharmacy practice and a pilot study [18].

## Factorial Survey Methodology

Factorial surveys are a useful method to study how healthcare professionals make real-life clinical decisions in response to complex situations [33-36], and have been previously applied to physicians [37-39], nurses [38-41], and pharmacists [27]. A factorial survey is a quasi-experimental design that differs from traditional surveys by the presence of factorial vignettes - a series of familiar scenarios where the respondent is asked to make judgements based on each scenario. The scenarios, which are derived from knowledge of clinical practice, share a common skeleton structure and include a set of embedded variables of interest to the research question. Plausible values for each variable within the vignette are randomly populated, creating a finite number of unique scenarios. The scenarios are allocated randomly to respondents, generating orthogonal or uncorrelated situations. Unlike static vignettes where we can only speculate what explains the responses, the factorial vignette design determines the independent effect of each included variable on the judgement made by the respondent to the scenario [33-36]. Accordingly, contextual factors such as time-pressures can be incorporated into the vignettes and be quantitatively evaluated for their influence on pharmacists’ clinical behaviours. Factorial surveys can also identify differences in responses to the scenarios due to characteristics at the respondent level. Thus, it is also possible to evaluate respondent’s beliefs, including medication monitoring attitudes, to test their influence on responses to the factorial vignettes [33-36].

## Factorial Vignette Skeleton and Vignette Factors

The initial factorial survey was developed and piloted on community pharmacy interns (n=121) completing the National Pharmacy Internship Programme (NPIP) during May and June 2016. The results and the feedback from this pilot survey informed the current survey and are reported elsewhere [18]. Briefly, in the pilot study each pharmacy intern completed five factorial vignettes of scenarios focused on repeat dispensing of antihypertensive medication to an older patient, reflective of pharmacy practice in Ireland. The initial vignette, included eight factors and was designed by academic pharmacists experienced in community pharmacy practice and was informed from a previous study [27]. Based on the quantitative results and qualitative feedback received from pharmacy interns during the pilot study, the original eight vignette factors were retained for the current survey with some modifications, and three new factors were added. Two of the new factors were based on qualitative feedback from the pilot study that indicated that further competing tasks exist in the form of administrative tasks (paperwork to claim reimbursement) and non-dispensary related patient interactions. Finally, an additional patient characteristic included in the final survey was a statement of the patient’s medication beliefs. It has been reported that community pharmacists are more likely to attribute non-adherence to technical or logistical issues rather than medication beliefs [42-45]. However, it is unknown whether community pharmacists are aware of the importance of patient medication beliefs as a determinant of medication adherence. Figure 2 details the final vignette and the eleven factors included in the vignettes are detailed in Table 1.

In response to five random factorial vignettes, respondents were asked to rate their likelihood to engage in three adherence monitoring behaviours:

1. Examine this patients dispensing records to assess adherence to antihypertensive medication over the previous months
2. Question this patient about their adherence to antihypertensive medication

1. Explore beliefs about antihypertensive medication that may influence this patient’s adherence

## Medication Monitoring Attitude Measure

The 15-item medication monitoring attitude measure (MMAM) was included in the questionnaire to evaluate attitudes, which may influence adherence monitoring behaviour [46]. The MMAM is designed to measure when and for whom pharmacists engage in medication monitoring and to assess their perceived role in medication monitoring. It consists of two subscales, with responses on a 6-point Likert scale, ranging from strongly disagree to strongly agree. The responses to each item are scored numerically (range 1-6, strongly disagree=1, strongly agree=6). The internal 7-item subscale contains items about pharmacist perception of role, motivation and responsibility (α=0.82). The external 8-item subscale focusses on busyness of the work environment and perceived patient acceptability of pharmacists engaging in medication monitoring (α=0.81). As the scale was developed for use in the US, the language used in the items were considered by a group of academic-based and clinically trained pharmacists at RCSI (n=5) and reworded to ensure suitability in the Irish context without changing the original meaning of the items (Appendix 1).

## Subjective norms and perceived behavioural control

Behavioural intention is theorised to also be influenced by subjective norms and perceived behavioural control [28]. To evaluate injunctive norms (IN), relevant referent individuals were identified from literature and informal discussions with academic-based community pharmacists. General practitioners (GPs) and patients have been identified as two important referent individuals [25] and following informal discussions, a single item was formulated for each (Table 2, IN1 and IN3). Additionally, an item evaluating whether as a pharmacist, respondents are expected to monitor antihypertensive adherence was included (Table 2, IN2). Descriptive norms (DN) capture whether important others perform the behaviours [47]. This was evaluated by asking respondents to rate whether other pharmacists perform the three adherence monitoring behaviours (Table 2, DN). Finally, self-efficacy (SE) was also assessed by asking respondents to rate the difficulty they would have in performing the three behaviours (Table 2, SE). For each of these items a 7-point semantic differential response scale with bipolar adjectives was employed as recommended in the development of TPB questionnaires [47].

## Pharmacist demographics and work environment

Respondent demographics such as gender, and professional experience information such as year of qualification, type and location of community pharmacy, number of hours worked per week, prescription activity and enhanced clinical services provided (24hr ambulatory blood pressure monitoring (ABPM)) were collected.

## Sample size and power calculation

Approximately 3,600 community pharmacists practice in Ireland (December 2016) and a sample of 347 was required to reach a statistically representative sample (95% confidence interval; 5% margin of error). Previous surveys of Irish community pharmacists observed a response rate of approximately 15% [48], thus a random sample of 2,315 community pharmacists would be required to obtain a statistically representative sample. In factorial surveys however, the vignette is considered the unit of analysis. A sample of 347 respondents would complete 1,735 randomly chosen vignettes from the 1,797,120 possible vignettes created for this survey. However, there are no well-established power analysis methods for hierarchical models in factorial surveys to determine whether 1,735 completed vignettes would provide adequate statistical power [37]. We took an approach using MLPowSim software package to estimate the power associated with each of the vignette factors for multilevel models (vignettes nested within respondents) which is described in Appendix 3. Based on this approach, assuming 350 respondents complete five vignettes each, all vignette factors are sufficiently powered (>80%) except for gender, number of prescription items and the telephone to collect later value. Rather than there being too few observations to test these factors’ influence, it may be that these factors do not influence responses to the scenario, as is the expected case for gender. Thus, based on the assumption of 350 respondents completing five vignettes each, and based on previous survey response rates, a sample of 2,315 pharmacists was considered sufficient. Permission was sought to obtain email addresses from the PSI for a simple random sample of 2,315 pharmacists from their registers who indicated on their annual registration that they practised in a community pharmacy role. However, following an application process the PSI provided a smaller simple random sample of email addresses for 1,543 pharmacists.

## Survey Administration

Using the mail merge function in MS Office 1,543 invitations to participate in the survey were sent via email in August 2017. The survey was self-administered online using a software system provided by Unipark Questback, Cologne, Germany. This software system was chosen due to its wildcard functionality, which enabled the importation of the factorial vignettes. The factorial vignettes were constructed using a randomisation procedure in Stata described by Auspurg & Hinz [36]. A simple random sample of 16,500 scenarios were drawn from the total vignette universe and 3,300 vignette decks were created, each containing five randomly allocated scenarios. This process creates 3,300 unique surveys, each a closed survey with a unique password. Each email address (n=1,543) obtained from the PSI was randomly matched to a unique survey and the password for each unique survey was embedded into the web link to the survey. These web links with embedded passwords were included in the invitation to participate and clicking the link provided direct access to a unique password protected survey. This method prevented multiple entries, as each password was only valid for a single survey, and prevented access to those outside the target sample who did not have valid passwords. The survey was open for 30 days with two reminders sent at 10 day intervals.

## Statistical Analysis

Descriptive statistics are presented to characterise respondent demographics and responses to the MMAM subscales, the subjective norms and self-efficacy questions. To test factors influencing responses to the factorial vignettes, multivariable multilevel linear regression modelling was performed to allow simultaneous consideration of vignette-level and pharmacist-level variation [35]. Level-1 variables for the multilevel regression include the vignette factors and level-2 variables include respondent factors (demographics, MMAM scores, subjective norms and self-efficacy responses). Firstly, a null model was tested to produce intra-class correlations (ICC) for responses to the vignette. ICCs reveal the proportion of variability attributable to respondent level variation for each of the three vignette responses. For the three vignette responses, separate multivariable multilevel linear regression modelling was performed, including all relevant factors for each theoretical influence of behaviour outlined in the conceptual framework (Figure 1). For regression analyses, MMAM-external scores were reverse-scored so that higher scores indicate environments that are more conducive to medication monitoring. To help identify factors which have the largest influence on adherence monitoring behaviour, standardised coefficients were also obtained by standardising all predictor variables (level-1 and level-2 variables) so that each predictor variable has a mean of zero and a standard deviation of one (i.e. z-transformation). To perform transformations, categorical variables were dummy coded. The resultant standardised coefficients represent a one unit change in vignette responses expected with a one standard deviation change in predictor variables.

Statistical modelling was performed using Stata version 14 (StataCorp College Station, Texas, USA).

# Results

## Response rate, demographics and work environment

In total, 1,543 email invitations were sent, and 368 survey responses were received. Of these 368 responses, eight did not provide consent to participate and 30 indicated during the eligibility check that they were not working as a community pharmacist in the Republic of Ireland. Seventy-two respondents partially completed the questionnaire, defined as failing to reach the final page of the questionnaire, and were excluded from the analysis. The final sample consisted of 258 respondents representing a response rate of 16.7%. Figure 3 outlines the number of respondents to the survey.

Approximately two-thirds of respondents were women, a third of respondents were qualified as a pharmacist for 15 years or longer, and over half indicated working as a support pharmacist. Similarly, over half reported working in independent pharmacies, predominantly located in non-rural areas, dispensing on average 225 items per day. Table 3 details respondent demographics and work environment.

## Medication Monitoring Attitudes

Summary scores for the MMAM subscales were calculated by obtaining the mean response to each item (range 1-6). The mean MMAM-internal score was 4.6 (*SD 0.7)*, indicating moderate agreement with the items in this scale. Thus on average, pharmacists tended to have a moderately positive attitude towards medication monitoring. The mean MMAM-external score was 3.2 *(SD 0.8)*, indicating neither agreement nor disagreement to the items on this scale. Thus, the respondents were neutral about conduciveness of their work environment- and patient acceptability towards medication monitoring.

## Subjective norms and self-efficacy

Overall respondents were neutral about whether GPs in their locality think that the respondent personally should assess adherence during repeat dispensing (mean 4.2 (*SD 1.5*)) (scale 1-7). In response to whether respondents perceive that they are expected to assess adherence during repeat dispensing, respondents tended to be positive (mean 5.5 (*SD 1.5*)), while similarly respondents tended to perceive that patients would approve of the respondent personally assessing their adherence (mean 5.2 (*SD 1.5*)).

In response to whether other pharmacists examine dispensing records to assess adherence, respondents tended to be positive (mean 5.2 (*SD 1.5*)), although in response to whether other pharmacists ask their patients questions about adherence, respondents were less positive (mean 4.8 (*SD 1.4*)). However, responses tended to be more neutral to the question about whether other pharmacists discuss medication beliefs that influence adherence with their patients (mean 4.4 (*SD 1.5*)).

Finally respondents tended to rate examining dispensing records to evaluate adherence to be an easy task (mean 5.5 (*SD 1.5*)), while asking patients questions about their adherence was rated to be less easy (mean 5.0 *(SD 1.5*)), and discussing medication beliefs with patients was rated to be less easy again (mean 4.7 (*SD 1.6*)).

## Factorial Vignette

### Examining dispensing records to assess adherence

The mean likelihood to examine the patient’s dispensing records to assess adherence to antihypertensive medication over the previous months was 6.4 (*SD 2.9, scale 1-10*) in response to the factorial vignettes (n=1,274)*.* An ICC of 0.59 was obtained from a null multilevel linear regression model indicating that 59% of the variation in responses is driven by respondent level characteristics. In the multivariable multilevel linear regression model (Table 4 - Model 1), pharmacists were more likely to examine dispensing records to assess adherence for each additional day the patient was late to collect the repeat prescription, if the pharmacy was fully staffed, while patient concerns and necessity beliefs appeared to increase the likelihood responses. An increasing number of patients waiting was negatively associated with likelihood responses. For the respondent-level factors, female pharmacists, pharmacists working with other pharmacists, working longer hours and stronger agreement that other pharmacists examine dispensing records to assess adherence were associated with higher likelihood responses. Providing 24 hr ABPM and stronger agreement that local GPs would approve pharmacists evaluating adherence were associated with lower likelihood responses.

### Questioning patients about adherence

The mean likelihood to ask the patient questions about their adherence was 5.3 (*SD 2.9*) in response to the factorial vignettes (n=1,274)*.* An ICC of 0.44 was obtained from a null multilevel linear regression model indicating that 44% of the variation in responses is driven by respondent level characteristics. In the multivariable multilevel linear regression model (Table 4 - Model 2), pharmacists were more likely to ask patients questions about their antihypertensive adherence for each additional day the patient was late to collect the repeat prescription, if the pharmacy was fully staffed, and if the patient previously expressed concerns or doubts about the need for antihypertensive medication. Vignette factors with a negative influence on likelihood responses were an increasing number of patients waiting and a longer time since antihypertensive treatment was initiated. Pharmacists with higher MMAM-internal scores and stronger agreement that other pharmacists ask their patients about antihypertensive adherence were more likely to ask their patients questions about adherence. Similar to the first outcome, providing 24hr ABPM was associated with lower likelihood scores while working with more pharmacists and working longer hours appears to be associated with higher likelihood responses.

### Explore beliefs about medication that influence adherence

The mean likelihood to discuss medication beliefs with patients was 4.7 (*SD 2.8*) in response to the factorial vignettes (n=1,269)*.* An ICC of 0.47 was obtained from a null multilevel linear regression model indicating that 47% of the variation in responses is driven by respondent level characteristics. In the multivariable multilevel linear regression model (Table 4 - Model 3), pharmacists were more likely to discuss medication beliefs with their patients for each additional day the patient was late to collect the repeat prescription, if the pharmacy was fully staffed, and if the patient previously expressed concerns or doubts about the need for antihypertensive medication. Vignette factors with a negative influence on likelihood responses were an increasing number of patients waiting, a longer time since antihypertensive treatment was initiated and if the patient did not present personally to collect the medication. Respondent factors that positively influenced likelihood to discuss medication beliefs were pharmacists with higher MMAM-internal scores and stronger agreement that other pharmacists discuss medication beliefs that influence antihypertensive adherence with patients.

# Discussion

## Principal Findings

In this factorial survey, responses to the MMAM, a validated and structured questionnaire to evaluate pharmacists’ attitudes towards medication monitoring, indicate that community pharmacists in the Republic of Ireland had moderately positive attitudes towards medication monitoring. However respondents’ were neutral about the busyness of the work environment and patient acceptability being conducive towards medication monitoring. In factorial vignette analysis, respondents’ attitudes towards medication monitoring were important influences as to whether they would monitor antihypertensive medication adherence by examining refill-patterns from pharmacy records, by asking patients questions about their adherence or their medication beliefs. Additionally, respondents’ normative beliefs, whether other pharmacists also performed these behaviours, were important influences. Furthermore, a number of contextual factors influenced respondents’ likelihood to perform the three adherence monitoring behaviours, including time-pressures and the number of days late the patient collected their repeat prescription.

## Pharmacist beliefs about adherence monitoring

A previous survey identified that Irish pharmacists were eager to provide enhanced services such as medication monitoring in community pharmacies [20]. In the current study, the MMAM was used to evaluate attitudes towards medication monitoring [46], and identified that pharmacists were moderately positive towards medication monitoring, however were neutral about conduciveness of their work environment- and patient acceptability towards medication monitoring. Few studies have previously evaluated community pharmacists’ beliefs specifically regarding medication adherence monitoring during repeat dispensing. A survey of US and Australian pharmacists similarly identified overall positive attitudes toward their role in adherence monitoring [27, 45]. In addition, Australian pharmacists reported that they believed that doctors and patients would also be positive about pharmacists monitoring adherence [45]. In contrast, respondents in the current study were less positive about GPs approving of them monitoring adherence. Similar experiences have been reported by some pharmacists implementing Medicines Use Reviews (MUR) and the New Medicines Service (NMS) in England, who perceived they were encroaching professional boundaries [21-23].

## Barriers and facilitators towards adherence monitoring

In response to the factorial vignettes, respondents were more likely to evaluate refill-adherence via dispensing records rather than interacting with patients to subjectively assess their adherence behaviour or their medication beliefs. This corresponds with previous findings that reviewing dispensing records was the most common strategy employed by pharmacists to identify non-adherence in comparison to asking questions regarding adherence behaviour or barriers to adherence [45]. Furthermore, the ICCs indicate that responses to examining dispensing records were influenced less by contextual factors than the two interactive behaviours requiring the pharmacist to ask the patient questions.

### Attitudes, normative beliefs and self-efficacy

Across the three adherence monitoring behaviours, the MMAM-external, did not influence responses. In contrast, a higher MMAM-internal score, which indicates higher motivation, role perception, and responsibility towards medication monitoring, had a strong positive effect on responses, although not statistically significant for the examination of dispensing records. Thus, pharmacists’ personal attitudes towards medication monitoring are more important influences than their perceptions of their environment in determining their likelihood to monitor adherence. Previous studies, have in contrast found both to be significantly associated with adherence- and medication monitoring [27]. However, a number of the MMAM-external items do not directly relate to adherence monitoring and other theoretical influences of behaviour including normative and control beliefs were not evaluated in these studies [28]. Positive descriptive norm beliefs in particular, had a strong positive effect on responses to the vignettes. Pharmacists reported higher intentions to perform each of the adherence monitoring behaviours in response to the vignettes if they perceived that other pharmacists performed these behaviours. In contrast, injunctive norms, and self-efficacy beliefs were not significant influences.

### Contextual influences

Across the three adherence-monitoring behaviours, a number of contextual factors embedded within the vignettes were consistently identified as barriers and facilitators to monitoring adherence. The patient’s refill-behaviour, modelled as the number of days early or late collecting their prescription, had a significant and relatively large effect on responses. This is consistent with our pilot study [18] and previous a US study [27]. This factor is a significant indication for pharmacists that a patient may have less than optimal adherence and a significant cue to evaluate patient adherence. Time-pressures had a negative influence on responses, specifically an increasing number of patients waiting for prescriptions and being short-staffed. Time-pressures have previously been highlighted as a barrier to medication and adherence monitoring [27, 45], and implementation of enhanced services and interventions in community pharmacy settings [15, 16, 21, 22].

Patients’ medication beliefs also influenced whether the pharmacist would question the patient about their adherence or medication beliefs. This contrasts previous findings that pharmacists tend to consider logistical reasons for non-adherence rather than motivations and beliefs [42-45]. Perceived patient medication beliefs however had a smaller influence on examining dispensing records and was underpowered due to overestimation of its predicted effect in power calculations. Similarly, a longer time on treatment negatively influenced whether pharmacists would question patients but not if they would examine dispensing records. This may reflect that generally, respondents were more inclined to perform this non-interactive behaviour over the two patient-interactive behaviours and that time since treatment initiation thus only becomes important when deciding to interact with patients. This corresponds with previous studies highlighting that pharmacists are more likely to counsel on new prescriptions rather than repeat prescriptions [49].

### Demographic influences

Respondent demographic factors did not have a consistent influence on likelihood to perform the three adherence behaviours. Female respondents and those who worked longer hours were more likely to examine dispensing records to evaluate adherence. However, gender did not influence the two adherence-monitoring behaviours that required the pharmacist to interact with the patient to discuss adherence behaviour and medication beliefs. The small positive influence of working longer hours may reflect pharmacists who are more familiar with their patients, although the patient-familiarity vignette factor did not influence responses. Working with more pharmacists appears to have a positive influence on responses, likely because of extra time to interact more with patients. This finding mirrors qualitative findings following the implementation of the NMS in England, which highlighted that having two pharmacists on duty was perceived to facilitate the implementation of the service [21]. The provision of 24hr ABPM in the current study appears to negatively influence responses, possibly reflecting an additional time-pressure associated with providing this service. This contrasts with previous findings on the provision of enhanced services in Australia [45], and England [21]. However, these settings differ from the Irish setting in terms of the funding of enhanced pharmacy services, where models of remuneration from the public health system exist. In Ireland patients privately pay for enhanced services such as 24 hr ABPM, which may be perceived by Irish pharmacists as supplementary rather than fundamental tasks.

## Strengths and limitations

A strength to this study is the use of a factorial survey methodology, which has high internal validity resulting from systematic variation of vignette variables in each situation, and randomly assigning each vignette to respondents. Factorial surveys can also achieve large sample sizes improving generalisability of findings [33-36]. However, factorial vignettes do not test actual behaviour; rather they assess behavioural intention in response to hypothetical situations. Behavioural intention has been shown to be a strong predictor of actual behaviour [28], and for clinicians is considered a valid proxy measure of actual behaviour [50]. Furthermore, the vignettes are hypothetical scenarios rather than real observations. However, they were designed by experienced community pharmacists and were intended to reflect everyday situations that pharmacists encounter, rather than abstract hypothetical scenarios. It is also possible that the scenarios do not reflect practice [35]. In this regard, we undertook piloting obtaining positive feedback from pharmacy interns on the realistic nature of the vignette scenarios. Additionally, in the current survey, respondents provided a high mean rating of 8.0 (*SD 2.1,* possible range 1-10) regarding the realistic nature of the scenario in relation to their practice. Social desirability bias is also a potential limitation with pharmacists overestimating responses to conform to ideals. It has been argued however that this form of bias may be less of an issue for factorial vignettes in comparison to real-life where respondents may be accountable for their decisions [34]. Additionally there are limitations to how the constructs of subjective norms and perceived behavioural control were measured. It would have been preferable to undertake qualitative work to elicit salient beliefs and to employ a number of different questions to obtain reliable and internally consistent measures of these constructs [47]. Finally, the desired target sample size to achieve a statistically representative sample was not achieved while also reducing statistical power. As a result, some of these factors may indeed have a small effect on the vignette responses, but a larger sample size would be required to confirm or reject this.

## Practice and research implications

Currently pharmacists in Ireland are not remunerated for providing adherence services and no structured adherence-monitoring program has been implemented in this setting. These findings could be used to inform the development of a structured pharmacy adherence-monitoring programme to underpin an adherence intervention. Firstly, pharmacists appear more likely to evaluate refill-adherence via dispensing records rather than interacting with patients, and this behaviour is less likely to be influenced by contextual factors including time-pressures such as other patients waiting and staffing levels. Priority should be given to identifying poor refill-adherence initially, and patients identified to have potential adherence issues could be followed-up with standardised questionnaires, to evaluate adherence behaviour and patient-specific barriers. However, the number of days late may not be readily accessible to pharmacists in the dispensing workflow [42]. To enable adherence monitoring in Irish community pharmacy, development of dispensing applications, which generate refill-adherence metrics and graphs, such as the Proportion of Days Covered and Group-based Trajectory Models is required.

Secondly, although contextual time-pressures had less of an influence on intentions to examine dispensing records, nonetheless the number of patients and staffing levels were significant negative influences. In addition, respondent level factors such as working with fewer pharmacists and the provision of an enhanced service (24hr ABPM) negatively influenced likelihood to examine dispensing records, perhaps reflecting the impact of time-pressures within the community pharmacy. Thus, the feasibility of implementing a structured adherence-monitoring programme in Irish community pharmacy may depend on extra resourcing or reorganisation of current workflow practices. In terms of extra resourcing, this could be funded similarly to other advanced services, such as the influenza vaccination programme, where pharmacists are reimbursed by the state health service per eligible patient vaccinated. However further research would be required to underpin the development and remuneration of such a service.

Finally, pharmacists’ beliefs regarding medication adherence monitoring influenced their likelihood to monitor adherence. In previous surveys, pharmacists reported that they were keen to perform enhanced services such as medication monitoring, and the community pharmacy has been advocated as an ideal location for such an intervention [20]. However, respondents to the current survey were moderately positive about medication monitoring and neutral regarding the conduciveness of the community pharmacy environment for medication monitoring. Addressing pharmacists’ behavioural and normative beliefs, could facilitate the implementation of a structured adherence-monitoring programme in community pharmacy. Further work is needed to develop training courses to facilitate an adherence-monitoring programme that could address these areas.

## Conclusion

Pharmacists potentially can play a role in identifying appropriate patients for adherence interventions and their reasons for non-adherence. This survey identified that positive pharmacist attitudes and normative beliefs can facilitate adherence monitoring within the current workflow; however contextual time-barriers may prevent adherence monitoring. Future research should consider these findings when designing a pharmacist-led adherence intervention to be integrated within current pharmacy workflow; alternatively novel working arrangements to facilitate adherence interventions within this setting should be considered.

# Abbreviations

ABPM: Ambulatory Blood Pressure Monitoring

DN: Descriptive norms

GPs: General practitioners

ICC: Intra-Class Correlations

IN: Injunctive Norms

MMAM: Medication Monitoring Attitude Measure

NPIP: National Pharmacy Internship Programme

PSI: Pharmaceutical Society of Ireland

RCSI: Royal College of Surgeons in Ireland

REC: Research and Ethics Committee

SE: Self-Efficacy

TPB: Theory of Planned Behaviour

# Declarations

## Ethics approval and consent to participate

Ethical approval for this study was granted by the Research and Ethics Committee at the Royal College of Surgeons in Ireland. Electronically declared informed consent was provided by participants prior to undertaking the survey (REC application 1356/2017).

## Consent for publication

Not applicable

## Availability of data and material

The datasets used and/or analysed during the current study are available from the corresponding author on reasonable request.

## Competing interests

Susan Smith is a member of the journal’s editorial board. The other authors have no conflicts of interest to declare.

## Funding

There is no funding to declare.

## Author contributions

PD, RMcD, SS, PG, GC were involved in the conception and design of the study. RMcD undertook the sample size calculations and provided statistical input. PD and GC undertook the acquisition, and analysis of the work. PD, SS, PG, GC interpreted the data. PD, SS, PG, GC drafted the manuscript. PD, RMcD, SS, PG, GC revised the manuscript and gave final approval of the version to be published. PD, RMcD, SS, PG, GC agree to be accountable for all aspects of the work in ensuring that questions related to the accuracy or integrity of any part of the work are appropriately investigated and resolved

## Acknowledgements

We thank the PSI for providing a sample of email-addresses from the register of pharmacists, which facilitated the administration of this survey.

# References

1. Chowdhury R, Khan H, Heydon E, Shroufi A, Fahimi S, Moore C, et al. **Adherence to cardiovascular therapy: a meta-analysis of prevalence and clinical consequences.** *Eur Heart J.* 2013;34(38):2940-8

2. Schroeder K, Fahey T, Ebrahim S: **Interventions for improving adherence to treatment in patients with high blood pressure in ambulatory settings**. *The Cochrane database of systematic reviews* 2004(2):CD004804.

3. Nieuwlaat R, Wilczynski N, Navarro T, Hobson N, Jeffery R, Keepanasseril A, Agoritsas T, Mistry N, Iorio A, Jack S *et al*: **Interventions for enhancing medication adherence**. *The Cochrane database of systematic reviews* 2014, **11**:CD000011.

4. Conn VS, Ruppar TM, Chase JA, Enriquez M, Cooper PS: **Interventions to Improve Medication Adherence in Hypertensive Patients: Systematic Review and Meta-analysis**. *Current hypertension reports* 2015, **17**(12):94.

5. Fuller RH, Perel P, Navarro-Ruan T, Nieuwlaat R, Haynes RB, Huffman MD: **Improving medication adherence in patients with cardiovascular disease: a systematic review**. *Heart* 2018, **104**(15):1238-1243.

6. Morrissey EC, Durand H, Nieuwlaat R, Navarro T, Haynes RB, Walsh JC, Molloy GJ: **Effectiveness and content analysis of interventions to enhance medication adherence and blood pressure control in hypertension: A systematic review and meta-analysis**. *Psychol Health* 2017, **32**(10):1195-1232.

7. Nguyen T-M-U, La Caze A, Cottrell N: **Validated adherence scales used in a measurement-guided medication management approach to target and tailor a medication adherence intervention: a randomised controlled trial**. *BMJ open* 2016, **6**(11):e013375.

8. Cutrona SL, Choudhry NK, Fischer MA, Servi AD, Stedman M, Liberman JN, Brennan TA, Shrank WH: **Targeting cardiovascular medication adherence interventions**. *Journal of the American Pharmacists Association : JAPhA* 2012, **52**(3):381-397.

9. Patton DE, Hughes CM, Cadogan CA, Ryan CA: **Theory-Based Interventions to Improve Medication Adherence in Older Adults Prescribed Polypharmacy: A Systematic Review**. *Drugs & aging* 2017, **34**(2):97-113.

10. Conn VS, Ruppar TM, Enriquez M, Cooper P: **Medication adherence interventions that target subjects with adherence problems: Systematic review and meta-analysis**. *Research in social & administrative pharmacy : RSAP* 2016, **12**(2):218-246.

11. Choudhry NK, Krumme AA, Ercole PM, et al.: **Effect of reminder devices on medication adherence: The remind randomized clinical trial**. *JAMA Intern Med* 2017, **177**(5):624-631.

12. Giardini A, Martin MT, Cahir C, Lehane E, Menditto E, Strano M, Pecorelli S, Monaco A, Marengoni A: **Toward appropriate criteria in medication adherence assessment in older persons: Position Paper**. *Aging Clin Exp Res* 2016, **28**(3):371-381.

13. **Public Survey - Attitudes to Pharmacy in Ireland** [<http://www.thepsi.ie/tns/news/latest-news/AttitudestoPharmacyinIreland.aspx>]

14. Costa E, Giardini A, Savin M, Menditto E, Lehane E, Laosa O, Pecorelli S, Monaco A, Marengoni A: **Interventional tools to improve medication adherence: review of literature**. *Patient preference and adherence* 2015, **9**:1303-1314.

15. De Simoni A, Mullis R, Clyne W, Blenkinsopp A: **Medicines optimisation in primary care: can community pharmacies deliver?** *The British journal of general practice : the journal of the Royal College of General Practitioners* 2012, **62**(601):398-399.

16. Cheema E, Sutcliffe P, Singer DR: **The impact of interventions by pharmacists in community pharmacies on control of hypertension: a systematic review and meta-analysis of randomised controlled trials**. *British journal of clinical pharmacology* 2014, **78**(6):1238-1247.

17. Kelly DV, Bishop L, Young S, Hawboldt J, Phillips L, Keough TM: **Pharmacist and physician views on collaborative practice: Findings from the community pharmaceutical care project**. *Canadian pharmacists journal : CPJ = Revue des pharmaciens du Canada : RPC* 2013, **146**(4):218-226.

18. Dillon P, Smith SM, Gallagher P, Cousins G: **Medication monitoring attitudes and perceived determinants to offering medication adherence advice to older hypertensive adults: a factorial survey of community pharmacy interns**. *The International journal of pharmacy practice* 2018.

19. **Future of Pharmacy Practice in Ireland: Meeting Patients' Needs**. In*.*: The Pharmaceutical Society of Ireland (PSI).

20. **Baseline Study of Community Pharmacy Practice in Ireland**. In*.*: The Pharmaceutical Society of Ireland (PSI).

21. Latif A, Waring J, Watmough D, Barber N, Chuter A, Davies J, Salema NE, Boyd MJ, Elliott RA: **Examination of England's New Medicine Service (NMS) of complex health care interventions in community pharmacy**. *Research in social & administrative pharmacy : RSAP* 2016, **12**(6):966-989.

22. Bradley F, Wagner AC, Elvey R, Noyce PR, Ashcroft DM: **Determinants of the uptake of medicines use reviews (MURs) by community pharmacies in England: A multi-method study**. *Health policy (Amsterdam, Netherlands)* 2008, **88**(2):258-268.

23. Lucas B, Blenkinsopp A: **Community pharmacists' experience and perceptions of the New Medicines Service (NMS)**. *The International journal of pharmacy practice* 2015, **23**(6):399-406.

24. (IPU) IPU: **IPU New Medicines Service( (NMS) Pilot 2017 Report**. In*.*; 2017.

25. Luetsch K: **Attitudes and attributes of pharmacists in relation to practice change – A scoping review and discussion**. *Research in Social and Administrative Pharmacy* 2017, **13**(3):440-455.e411.

26. Rosenthal M, Austin Z, Tsuyuki RT: **Are Pharmacists the Ultimate Barrier to Pharmacy Practice Change?** *Canadian Pharmacists Journal / Revue des Pharmaciens du Canada* 2010, **143**(1):37-42.

27. Witry MJ, Doucette WR: **Factors influencing community pharmacists' likelihood to ask medication monitoring questions: A factorial survey**. *Research in Social and Administrative Pharmacy* 2015, **11**(5):639-650.

28. Ajzen I: **The theory of planned behavior**. *Organizational Behavior and Human Decision Processes* 1991, **50**(2):179-211.

29. Armitage CJ, Conner M: **Efficacy of the Theory of Planned Behaviour: a meta-analytic review**. *The British journal of social psychology* 2001, **40**(Pt 4):471-499.

30. Perkins MB, Jensen PS, Jaccard J, Gollwitzer P, Oettingen G, Pappadopulos E, Hoagwood KE: **Applying theory-driven approaches to understanding and modifying clinicians' behavior: what do we know?** *Psychiatric services (Washington, DC)* 2007, **58**(3):342-348.

31. Godin G, Belanger-Gravel A, Eccles M, Grimshaw J: **Healthcare professionals' intentions and behaviours: a systematic review of studies based on social cognitive theories**. *Implementation science : IS* 2008, **3**:36.

32. Grol R, Wensing M: **What drives change? Barriers to and incentives for achieving evidence-based practice**. *The Medical journal of Australia* 2004, **180**(6 Suppl):S57-60.

33. Rossi PH, Nock SL: **Measuring social judgments: The factorial survey approach**: SAGE Publications, Incorporated; 1982.

34. Taylor BJ: **Factorial Surveys: Using Vignettes to Study Professional Judgement**. *British Journal of Social Work* 2006, **36**(7):1187-1207.

35. Jasso G: **Factorial survey methods for studying beliefs and judgments**. *Sociological Methods and Research* 2006, **34**(3):334-423.

36. Auspurg K, Hinz T: **Factorial Survey Experiments**. Thousand Oaks, California: Sage; 2015.

37. Muller-Engelmann M, Krones T, Keller H, Donner-Banzhoff N: **Decision making preferences in the medical encounter--a factorial survey design**. *BMC Health Serv Res* 2008, **8**:260.

38. Bos-Touwen ID, Trappenburg JC, van der Wulp I, Schuurmans MJ, de Wit NJ: **Patient factors that influence clinicians' decision making in self-management support: A clinical vignette study**. *PloS one* 2017, **12**(2):e0171251.

39. Hill L, McIlfatrick S, Taylor BJ, Jaarsma T, Moser D, Slater P, McAloon T, Dixon L, Donnelly P, Stromberg A *et al*: **Patient and Professional Factors That Impact the Perceived Likelihood and Confidence of Healthcare Professionals to Discuss Implantable Cardioverter Defibrillator Deactivation in Advanced Heart Failure: Results From an International Factorial Survey**. *The Journal of cardiovascular nursing* 2018.

40. Ludwick R, Wright ME, Zeller RA, Dowding DW, Lauder W, Winchell J: **An improved methodology for advancing nursing research: factorial surveys**. *ANS Advances in nursing science* 2004, **27**(3):224-238.

41. Brenner M, Drennan J, Treacy MP, Fealy GM: **An exploration of the practice of restricting a child's movement in hospital: a factorial survey**. *Journal of clinical nursing* 2015, **24**(9-10):1189-1198.

42. Witry MJ, Doucette WR: **Community pharmacists, medication monitoring, and the routine nature of refills: a qualitative study**. *Journal of the American Pharmacists Association : JAPhA* 2014, **54**(6):594-603.

43. Cocohoba J, Comfort M, Kianfar H, Johnson MO: **A qualitative study examining HIV antiretroviral adherence counseling and support in community pharmacies**. *Journal of managed care pharmacy : JMCP* 2013, **19**(6):454-460.

44. Witry M, Parry R, McDonough R, Deninger M: **Analysis of medication adherence-related notes from a service-oriented community pharmacy**. *Research in social & administrative pharmacy : RSAP* 2017.

45. Mansoor SM, Krass I, Costa DS, Aslani P: **Factors influencing the provision of adherence support by community pharmacists: A structural equation modeling approach**. *Research in social & administrative pharmacy : RSAP* 2015, **11**(6):769-783.

46. Witry MJ, Wesely PM, Goedken AM, Ernst EJ, Sorofman BA, Doucette WR: **Development of a medication monitoring attitude measure using a mixed methods item development process**. *The International journal of pharmacy practice* 2016, **24**(1):49-59.

47. Fishbein M, Ajzen I: **Predicting and Changing Behavior: The Reasoned Action Approach**: Psychology Press; 2010.

48. Redmond P, Carroll H, Grimes T, Galvin R, McDonnell R, Boland F, McDowell R, Hughes C, Fahey T: **GPs' and community pharmacists' opinions on medication management at transitions of care in Ireland**. *Family practice* 2016, **33**(2):172-178.

49. Puspitasari HP, Aslani P, Krass I: **A review of counseling practices on prescription medicines in community pharmacies**. *Research in social & administrative pharmacy : RSAP* 2009, **5**(3):197-210.

50. Eccles MP, Hrisos S, Francis J, Kaner EF, Dickinson HO, Beyer F, Johnston M: **Do self- reported intentions predict clinicians' behaviour: a systematic review**. *Implementation science : IS* 2006, **1**:28.

# Figure Legends

***Figure 1 Conceptual model outlining the possible factors influencing pharmacists’ adherence monitoring behaviour during repeat dispensing.***

***Detailed legend for figure 1: The blue circles represent the constructs of the Theory of Planned Behaviour (TPB), while the white circles represent the variables measured in this survey mapped onto the relevant construct of the TPB.***

***Figure 2 Final vignette with the labels of the factors which were varied systematically highlighted in red. The values for each of the labels are detailed in table 1.***

***Figure 3 Flowchart of respondent numbers to survey***

# *Additional files*

***Appendix 1***

- ***Questionnaire***
- ***The questionnaire completed by survey respondents***

***Appendix 2***

- ***Study information leaflet***

***Appendix 3***

- ***Sample size***
- ***Detailed description of procedure to estimate sample size for the survey***
