# Supplementary material for: Determinants of intentions to monitor antihypertensive medication adherence in Irish community pharmacy: a factorial survey
Source: BMC Fam Pract. 2019 Sep 13;20:131. doi: 10.1186/s12875-019-1016-6 (PMC6744667; doi:10.1186/s12875-019-1016-6)
Supplement: Supplementary file 3 — Sample size. Detailed description of procedure to estimate sample size for the survey (ZIP 213 kb) [file 12875_2019_1016_MOESM3_ESM.zip › Sample size calcR3.docx]

## Sample size

Approximately 3,600 community pharmacists practice in Ireland (December 2016) and a sample of 347 was required to reach a statistically representative sample (95% confidence interval; 5% margin of error). Previous surveys of Irish community pharmacists observed a response rate of approximately 15% [47], thus a random sample of 2,315 community pharmacists would be required to obtain a statistically representative sample. In factorial surveys, the vignette is considered the unit of analysis. A sample of 347 respondents would complete 1,735 randomly chosen vignettes from the 1,797,120 possible vignettes. However there are no well-established power analysis methods for hierarchical models in factorial surveys [37]. An approach using MLPowSim software package was used to estimate the power associated with each of the vignette factors for multilevel models (vignettes nested within respondents) and is described in Appendix 2. As the software was unable to handle more than 13-14 factors, respondent level factors could not be included in the power calculation. Statistical significance was set at 5% on the basis of a 2-sided design based test. In order to perform the power calculations the following were required from the pilot study; the intra-cluster correlation **(**ICC=0.3), the estimated effects of the eight vignette factors, and the mean and variance of each of the vignette factors. For the three new factors, the estimated effect sizes of the month-end claim and patient query factors was set at -0.8, while the effect of the non-blank levels of the patient beliefs factor was set at +0.3. The means and variances for these new factors were assumed using comparable factors from similar factor types from the pilot study (gender, familiarity). Orthogonality of the predictors was assumed based on the factorial randomisation procedure. Categorical variables were recoded to dummy variables and length of time on treatment was treated as a continuous variable. Furthermore, for the familiarity factor, power was computed for the original binary value rather than its modified factor. Table 4 details the estimated power for the eleven vignette factors, assuming 350 respondents complete five vignettes each. All vignette factors are sufficiently powered (>80%) except for gender, number of prescription items and the telephone to collect later value. Rather than there being too few observations to test these factors’ influence it may be that these factors do not influence responses to the scenario, as is the expected case for gender.

Table. The power associated with each of the vignette factors in the multilevel model

| **Vignette Factor** | Power |
| --- | --- |
| **Gender** | 0.32 |
| **Familiarity** | 0.98* |
| **Month-end Claim** | 1.00 |
| **Waiting/Collecting**  *Collect later*  *Daughter Collect* | 0.12  1.00 |
| **Number of Rx Items** | 0.18 |
| **Days Early/Late** | 1.00 |
| **Time since diagnosis** | 1.00 |
| **Medication Beliefs**  *Doubts about necessity*  *Concerns about medication* | 0.80  0.83 |
| **Patients waiting** | 1.00 |
| **Staff-levels** | 1.00 |
| **Patient Query** | 1.00 |

The power calculation was performed in MLPowSim and is based on 350 respondents completing five random vignettes each. Between and within respondent variances, estimated effects of each vignette factor and the mean and variance of each predictor were assumed based on the pilot study. *Computed for original binary factor.
